# Supplementary material for: Complexome profiling of the Chlamydomonas psb28 mutant reveals TEF5 as an early PSII assembly factor
Source: Plant Cell. 2025 Mar 17;37(6):koaf055. doi: 10.1093/plcell/koaf055 (PMC12164590; doi:10.1093/plcell/koaf055)
Supplement: koaf055_Supplementary_Data [file koaf055_supplementary_data.zip › psb28_interactive.zip!/plots/intensity/qualityPlots/peptides/CDC48_Cre06.g269950.t1.2.html]

### Cre06.g269950.t1.2

Trivial name: CDC48  
SeqCoverage [median]: 3.40  
Mass: 90.23 kDa  
  
 

| Sequence | MissedCleavage | Mass [Da] | wtIntensity | muIntensity | Score |
| --- | --- | --- | --- | --- | --- |
| IVSQLLTLMDGLK | 0 | 1429.82 | 1006.74 | 0.00 | 126.76 |
| NMKLDEDVNLEAISR | 1 | 1745.86 | 0.00 | 528.62 | 44.20 |
|  |  |  | 1006.7 | 528.6 |  |
